# P-1568. Reevaluating Our Urine Culture Clinical Support Tool: Do I Really Need “Other” as an Option?

**DOI:** 10.1093/ofid/ofae631.1735

**Published:** 2025-01-29

**Authors:** Mina Said, Vanessa Kung, Abigail Beck, Kristin Nagaro, Graham M Snyder, Elise Martin, Deanna Buehrle

**Affiliations:** UPMC, Pittsburgh, Pennsylvania; VA Pittsburgh Healthcare System, Pittsburgh, Pennsylvania; VA Pittsburgh Healthcare System, Pittsburgh, Pennsylvania; VA Pittsburgh Healthcare System, Pittsburgh, Pennsylvania; University of Pittsburgh, Pittsburgh, PA; VA Pittsburgh Healthcare System, Pittsburgh, Pennsylvania; VA Pittsburgh, Pittsburgh, Pennsylvania

## Abstract

**Background:**

One strategy to reduce non-indicated urine cultures and asymptomatic bacteriuria is an electronic health record tool requiring providers to select an indication, but limited data exist on such tool’s optimal indications and if “other” is a necessary option.

**Figure 1:**
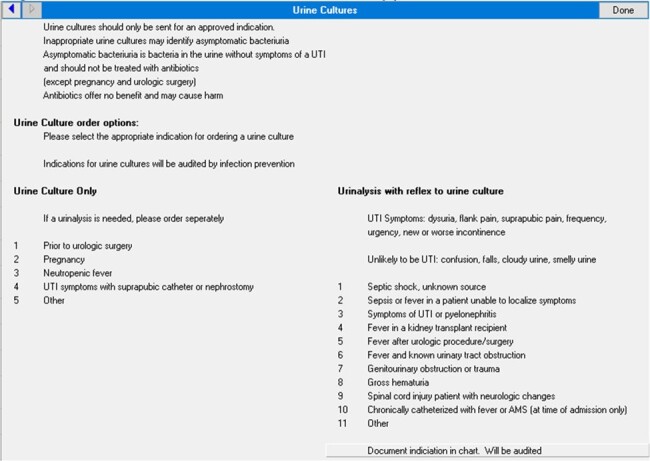
Urine culture clinical decision support tool with indication selection at time of ordering.

**Methods:**

In 1/2023, our VA facility developed a urine reflex/culture ordering clinical decision support (CDS) tool requiring an approved indication selection with each order (Figure 1), and audit/feedback emails for non-indicated orders were added in 7/2023. We performed a quality improvement evaluation of the CDS tool’s selected indications to understand ordering trends including appropriate orders (medically indicated with matched chart reviewed clinical scenario), wrong selection (medically indicated but selected indication did not match chart reviewed clinical scenario), non-indicated (no appropriate indication on chart review), and all “other”. We performed a chart review of 1063 orders (44%) from 1/17/23 - 12/30/23 and compared the CDS tool selections to chart review. We analyzed the percentage and distribution of appropriate, wrong selection, and non-indicated orders, and separately analyzed all “other” selections.

**Table 1:**
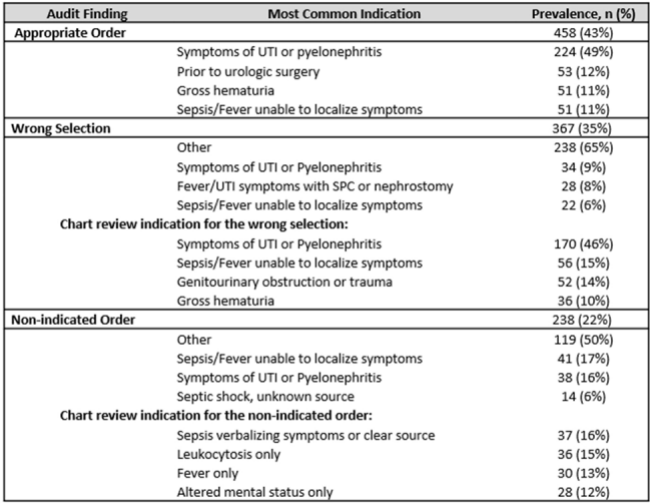
Proportion of selected indications deemed appropriate, wrong selection, and non-indicated, with the most common reasons for each.

UTI: Urinary tract infection, SPC: Suprapubic catheter

**Results:**

Of all urine reflex/culture orders audited, 43% were appropriately ordered, wrong selections were detected in 35%, while 22% were medically non-indicated (Table 1). The indication “other” was selected 357 times (33% of all orders) and only 1 “other” selection was considered appropriate (Table 2). 67% of “other” were selected instead of a medically indicated choice available on the order set. 33% of “other” were selected when a urine culture was not medically indicated, such as leukocytosis (16%), altered mental status without infectious symptoms (14%), or fever without urinary symptoms (11%). “Other” was also selected in 14 patients when ordering providers wanted a different urine test and did not need a culture.

**Table 2:**
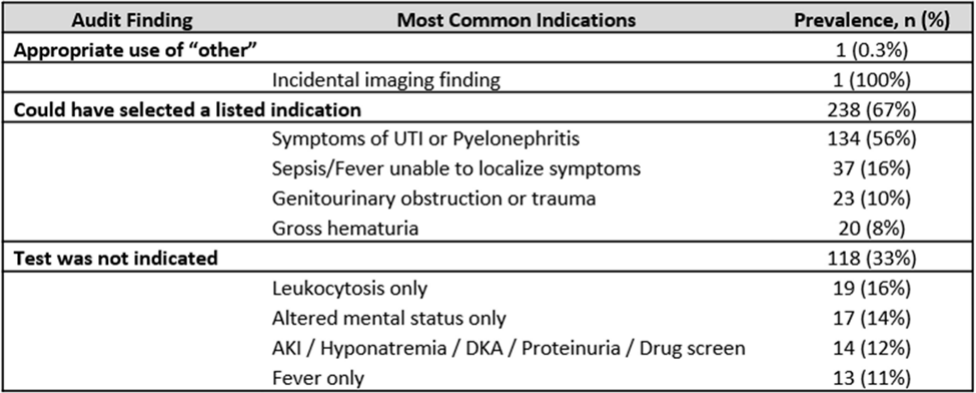
Assessment of indications when the ordering provider selected “other” for a urine culture or a urinalysis with reflex to culture.

UTI: Urinary tract infection, AKI: Acute kidney injury, DKA: Diabetic keto-acidosis

**Conclusion:**

While CDS tools may be effective in reducing non-indicated urine cultures, wrong selection and bypassing the order tool were common at our facility. Selecting “other” was a common source of bypassing the tool and for medically non-indicated urine cultures. Further research is necessary on whether removing “other” helps to reduce non-indicated urine reflex/cultures.

**Disclosures:**

**Graham M. Snyder, MD, SM**, Infectious Diseases Connect: Advisor/Consultant

